# Being both a grandmother and a health worker: experiences of community-based health workers in addressing adolescents’ sexual and reproductive health needs in rural Zambia

**DOI:** 10.1186/s12889-024-18685-6

**Published:** 2024-05-03

**Authors:** Chama Mulubwa, Joseph Mumba Zulu, Anna-Karin Hurtig, Isabel Goicolea

**Affiliations:** 1 School of Public Health, University of Zambia, P.O. Box 50110, Lusaka, Zambia; 2 Department of Epidemiology and Global Health, Umeå University, Umeå, SE Sweden; 3 Centre for Infectious Disease Research in Zambia (CIDRZ), P.O Box 34681, Lusaka, Zambia

**Keywords:** Adolescents, Community-based, Health workers, Sexual and reproductive health and rights, Zambia

## Abstract

**Introduction:**

Community-based health workers (CBHWs) possess great potential to be the missing link between the community and the formal health system for improving adolescents’ access to sexual and reproductive health and rights (SRHR) information and services. Yet, their role in addressing adolescents’ SRHR within the context of the community-based health system has received very little attention. This paper analyses how CBHWs experience and perceive their role in addressing adolescents’ SRHR needs in rural Zambia, including the possible barriers, dilemmas, and opportunities that emerge as CBHWs work with adolescents.

**Methods:**

Between July and September 2019, we conducted 14 in-depth interviews with 14 community-based health workers recruited across 14 different communities in the central province of Zambia. The interviews were focused on eliciting their experiences and perceptions of providing sexual and reproductive health services to adolescents. Charmaz’s grounded theory approach was used for the analysis.

**Results:**

We present the core category “being both a grandmother and a CBHW”, which builds upon four categories: being educators about sexual and reproductive health; being service providers and a link to SRHR services; being advocates for adolescents’ SRHR; and reporting sexual violence. These categories show that CBHWs adopt a dual role of being part of the community (as a grandmother) and part of the health system (as a professional CBHW), in order to create/maximise opportunities and navigate challenges.

**Conclusion:**

Community-based health workers could be key actors providing context-specific comprehensive SRHR information and services that could span all the boundaries in the community-based health system. When addressing adolescents SRHR, playing dual roles of being both a grandmother and a professional CBHW were sometimes complimentary and at other times conflicting. Additional research is required to understand how to improve the role of CBHWs in addressing adolescents and young people’s sexual and reproductive health.

**Supplementary Information:**

The online version contains supplementary material available at 10.1186/s12889-024-18685-6.

## Introduction

Access to comprehensive sexual and reproductive health and rights (SRHR) information and services is fundamental to ensure that adolescents enjoy their sexuality and achieve the highest attainable standards of health [1, 2]. In many countries, policies are available to guide SRHR service delivery to adolescents and young people, yet adolescents continue to face specific disadvantages when it comes to accessing SRHR services and information. Some of these disadvantages arise due to social stigma and discrimination surrounding adolescents’ sexual health, lack of comprehensive knowledge, privacy concerns, social norms that hinder discussion about SRHR and the requirement of parental consent for adolescents to access certain SRHR services [3–6]. 

In the last few decades, Zambia has made progress towards addressing adolescents’ SRHR through the formal health system. This includes development of the national policies and strategies specific to adolescents, such as the National Adolescent Sexual and Reproductive Health framework, establishments of youth friendly services and comprehensive sexuality education [6, 7]. However, meeting adolescents’ SRHR needs remains challenged by several factors, including low levels of knowledge among adolescents on available SRHR services [8], high levels of perceived stigma [9], unfriendly service provision by health workers, long distances to health facilities, and a lack of financial means to cover the direct and indirect costs related to SRHR services, to name just a few. Some of these challenges can be eliminated by taking a community-based approach that incorporates other key actors beyond the formal health system in the provision of comprehensive SRHR information and services [10–12].

## Adolescents’ SRHR information and services in Zambia’s community-based health system

Community-based health systems (CBHSs) are comprehensive structures that support healthcare within communities, and link together the formal health system, local actors (such as parents and community health workers) and civil society organisations [13]. In Zambia, these local actors work together with the health centres located at the lowest level of the formal health system. Of key relevance within CBHSs, especially in low- and middle-income countries, are community-based health workers (CBHWs).

Community-based health workers are community members who serve as volunteer or paid frontline healthcare professionals, usually with minimal or no formal training. In many countries, CBHWs have been used to provide basic health services within their communities as a way of increasing human resources dedicated to health [14–20]. The services provided by CBHWs are diverse across countries. Some of the common ones include being part of local family healthcare teams, providing services relating to maternal and child health, the mobilisation of health services, and the provision of SRHR services, including HIV [21, 22]. 

Several challenges affecting the delivery of services by CBHWs have been documented in countries across Africa, including Zambia. Within the health system, these challenges involve problems such as a lack of proper supervision for CBHWs, either limited or absent compensation for their work, health workers disregarding the clients referred to healthcare facilities by CBHWs, and the inability to consistently supply CBHWs with the necessary tools and supplies needed for them to effectively carry out their duties [11, 23, 24]. At the community level, CBHWs struggle to build trusting and healthy relationships with the community and various stakeholders. In addition, CBHWs experience challenges navigating between the health system’s expectations, the various community-based structures and stakeholders they serve, and implementing health services while representing the voice of the community and promoting community ownership of SRHR programmes [21]. 

In the Zambian CBHS, CBHWs work in partnership with various stakeholders to deliver SRHR services targeting young people and adolescents through various settings, including health facilities, schools, home settings, community spaces, and the police station (used for support in cases of gender-based violence) [25]. Specific SRHR services provided by CBHWs in Zambia to communities including young people involve the provision of SRHR information, counselling on various SRHR issues (i.e.,HIV, maternal health, pregnancies and early marriages), the distribution of condoms, and linkage the distribution of condoms, and linkage to HIV, contraceptives, and antenatal services for pregnant girls at the local health facility [11, 26]. 

Despite the potential of CBHWs to be a source of reliable health-related information (including SRHR information), the delivery of SRHR information and services for young people continue to be affected by socio-cultural norms, logistical challenges, such as distance, and beliefs that discussing sexuality issues with adolescents is a ‘taboo’ [27, 28]. In addition, CBHWs are not the only (and may not even be the most widely used) source of information in relation to SRHR: adolescents also have other sources of SRHR information, such as peers or relatives. In Zambia, an important traditional figure in the provision of SRHR information is that of grandparents, who are socially trusted by both adolescents and parents. Based on the existing literature, grandparents could be described as socially accepted, ‘non-threatening’ older people residing within the community with whom adolescents have (in)formal ties [29, 30]. 

Within the CBHS, CBHWs possess great potential to be the missing link between the community and the formal health system because they can work with adolescents at community level and also link them to essential SRHR services at the local health facility. This is especially relevant in countries like Zambia with significant gaps in human resources for health and facing a high burden of SRHR challenges among adolescents [5, 24, 31]. For example, a few studies conducted in Zambia showed that CBHWs can successfully worked with key stakeholders to deliver SRHR among adolescents [11, 26, 32]. For example, a few studies conducted in Zambia showed that CBHWs can successfully work with key stakeholders to deliver SRHR among young people [11, 26, 32]. Yet, while there is widespread evidence of CBHWs’ roles in providing universal healthcare and other SRHR services related to HIV and maternal health, their experiences and roles in addressing adolescents’ SRHR within the context of the CBHS has received far less attention. Many of the studies described earlier have focused on describing the role of CBHWs generally, without consideration of the CBHS context. Similarly, there exists little research on the possible barriers, dilemmas, and opportunities that emerge as CBHWs work to address adolescents’ SRHR needs and how they navigate through the process. Therefore, the aim of this study is to analyse how CBHWs experience and perceive their role in addressing adolescents’ SRHR needs in rural Zambia, including the barriers, dilemmas, and opportunities they face in fulfilling this role. The findings developed in this study could be the first step to understanding the barriers and opportunities of using CBHWs to enhance SRHR services within the framework of the community health system. These findings could be useful to policymakers and program implementers of SRHR programs aimed adolescents in Zambia and other comparable contexts within Sub-Saharan Africa.

## Conceptual framework: boundary spanning and discretion

We build upon the theoretical concept of boundary spanning to understand how CBHWs manipulate and bend the borders between their community and the healthcare system, as well as other sectors like education and law enforcement. This includes their role in bridging gaps between various groups of actors within these systems. [29]. Boundary spanning refers to the way in which individual actors (referred to as ‘boundary spanners’) work within settings that involve operating beyond their own structural and social or cultural groups to deliver public information and services [30, 33]. Boundary spanning involves establishing, transgressing, maintaining, negotiating, and dissolving boundaries. Hence, previous studies have described boundary spanners as ‘lynchpins’, ‘connectors’, ‘brokers’, and ‘gatekeepers’ [33–35]. 

In addition, we use Lipsky’s theory relating to street-level bureaucrats, and especially the concept of discretion, in order to further understand how CBHWs navigate the challenges of implementing health services and the (possible) relative power that they use to shape interventions and policies [29, 36]. The exercise of discretion is a critical component of the theory because it offers street-level bureaucrats the leeway to adapt policy, rules, and regulations during the implementation process [36, 37]. In many cases, this discretion is exercised as a way of navigating the various challenges and barriers encountered during their work. In this paper, we align our findings with those of other studies that highlight how CBHWs navigate inadequate resources, a growing demand for their services, and the political and/or social climate [11, 38]. 

## Methodology

### Study setting

This paper is part of a broader project analysing SRHR interventions for adolescents in randomly selected communities in rural Zambia [39]. We purposively selected 14 CBHWs across 14 communities in Chibombo and Kapiri districts of Zambia. The two districts were conveniently selected as the continuation to the previous work we have done with community health workers. The selected districts were also characterised by high proportion of teenage pregnancies and early marriages reported during the implementation of the main project. Alongside their ordinary work as CBHWs, all of our participants had participated in an SRHR intervention to support adolescent girls, working in collaboration with teachers to establish or strengthen adolescent clubs. These aimed to increase knowledge of SRHR, including modern contraceptives, and to promote contraceptive use among adolescent girls and boys [39]. As part of this intervention, the CBHWs also coordinated community and parental meetings for a period of two years.

### Study participants and recruitment

Data was collected between July and September 2019. To ensure a diversity of perspectives and communities, we extended invitations all the 18 potential CBHWs, from 18 distinct communities across Chibombo and Kapiri districts of Zambia where the broader SRHR project had been implemented. The invitations to participate in the interviews were conducted via phone. Out of the 18 CBHWs eligible for the interviews, 14 agreed to participate and were interviewed. Two of the participants belonged to the cadre of CBHWs referred to as Community Health Assistants, meaning that they had undertaken 12 months’ formal training and received a monthly stipend from the government, while the rest of the participants were ‘ordinary’ community health workers without formal training who worked on a volunteer basis. All of the 14 CBHWs who participated in this study were women. Four were aged between 41 and 50 years and 10 were aged between 50 and 64 years. All the participants had been working as CBHWs for more than 10 years.

### Data collection

All the interviews were conducted face to face by the first author (CM), in a mixture of Bemba and Nyanja, the languages spoken locally by both the interviewer and the participants. We used the interview guide specifically developed for this study (Appendix 1). The interviews focused on discussing the experiences of CBHWs and the potential roles that they play in addressing adolescents’ sexual and reproductive health and rights (SRHR) needs within the setting of the community-based health system. Each interview lasted for approximately 40 to 60 min and was digitally recorded. Alongside data collection, the co-authors (CM, JMZ, AKH, and IG) held frequent data analysis meetings to discuss candidate categories. Information from the preliminary analysis meetings helped in shaping subsequent interviews.

### Data analysis

All the interviews were transcribed verbatim and afterwards translated into English, so that the entire team of authors could read them. Both the transcribed and translated transcripts were then exported to NVIVO 11 to support the analysis. Charmaz’s constructivist grounded theory approach was used [40], which was linked to the iterative process of data collection and analysis described above. Once the transcripts were available, they were read and re-read and then coded line by line by the first author (CM). Initial codes were then shared with the co-authors (JMZ, AKH, and IG) for discussion. Afterwards, focused coding was performed. Preliminary categories were then developed, which later became more refined. The coding and development of categories was conducted in the original languages (that the first CM and one of the other authors JMZ are fluent in). To ensure the correct meaning and quality of the transcripts, an expert language translator was used to translated sections of the interviews. In addition, translated transcripts were later back translated to English by the first author.

To enhance credibility, interactive and constant discussions coupled with continuous note-taking were used throughout the entire analysis. This process continued until the core category and four sub-categories had been constructed [41]. 

### Ethical considerations

Ethical approval for this study was granted by the Excellence in Research Ethics and Science (ERES) committee in Lusaka, Zambia (approval number 2018-Jan-007). Written consent was obtained from each CBHW prior to the commencement of the interview. To ensure confidentiality, all data was anonymised by removing all personal identifiers.

### Findings

In this section, we present the core category “being both a grandmother and a CBHW”, which builds upon four sub-categories: (i) being educators about sexual and reproductive health; (ii) being service providers and a link to SRHR services; (iii) being advocates for adolescents and their SRHR; and (iv) reporting sexual violence.

### Being both a grandmother and a CBHW

The core category conveys how CBHWs experienced their role in addressing adolescents’ SRHR, which often involved playing two complementary (and sometimes contradictory) roles: being a professional CBHW and a grandmother. Due to their role of providing SRHR information and counselling, and partly because of their age and gender (middle-aged and older women), most CBHWs had earned the title of ‘grandmother’. From this position of grandmother, it was socially acceptable for them to openly discuss issues related to sexuality with young people. This position of grandmother was thus portrayed as critical in reducing the communication gap between CBHWs and adolescents during SRHR conversations. As one participant described it:

*When we’re teaching the boys and girls, we tell them to be free, be open and to ask us any questions they have. They ask, “grandmother what about so and so?”…even about sexual things. So, they’re free and ask us questions – KII 1*.

In addition, the role of professional CBHW granted them trust and legitimacy to discuss SRHR issues at a ‘professional’ level. Combining the dual role of grandmother and professional CBHW was thus enabling not only when dealing with adolescents but also when providing SRHR education to parents and community members. Combining this dual role sometimes promoted openness, trust, and care, as we further elaborate in the categories below.

At other times, CBHWs felt conflicted about combining the two roles, and this combination of roles could bring them into conflict with the community. Fulfilling the grandmother’s role generally focused on promoting socially and culturally ‘acceptable’ norms of adolescent sexuality, such as prescribing abstinence for unmarried adolescents. This sometimes conflicted with fulfilling the professional CBHW’s role, since the latter also includes providing comprehensive information about contraceptives. The four categories below represent how CBHWs experienced this dual role and navigated the challenges it brought in relation to being educators about sexual and reproductive health, being service providers and a link to SRHR services, being advocates for adolescents and their SRHR, and reporting sexual violence.

#### Being sexual and reproductive health educators

CBHWs considered that improving knowledge through education was a key role, and that they played this role through organising youth clubs for adolescents, and community meetings for parents and community members. Providing SRHR education for adolescents, parents, and community members was seen as a way to end SRHR problems such as adolescent pregnancies, child marriages, and sexual violence. Despite the anticipated benefits of SRHR education, actually conducting it sometimes brought about opposition. Discussing topics related to contraceptive use was met with disapproval from some parents, community members, and religious groups. One participant explained how some community members had reacted negatively to the idea of discussing contraceptives with adolescents:

*I: How do parents and community members react when they hear you teaching young people about SRHR issues such as the use of condoms and contraceptives?*

*R: Just there, the first meeting we had in the community, they [referring to parents and religious leaders] were very angry, especially those from Churches, especially the church where I go to … “that one, that (grand)mother [referring to the CBHW] is giving children condoms”. I didn’t do anything; I was, like, scared because they were very angry at church. Because, you know, at church because of that they find you at fault. – KII 7*.

The need to manage this opposition when educating adolescents and parents led CBHWs to draw upon their grandmother role. Most of them experienced the grandmother role as enabling them to gain the trust of parents more easily. Based on that trust, some parents would invite CBHWs to educate adolescents about menstrual hygiene and pregnancy prevention, or other SRHR discussions that they may have otherwise considered ‘inappropriate’ to discuss with their children, as conveyed in the quotation below:

*Yes, sometimes the parents would just send them [referring to adolescents] to you… you people [CBHWs], “I’ve sent my child; she seems to be sleeping with men, please talk to her”. And then as a grandmother you call the girl and talk to her… – KII 5*.

The participants argued that this trust shown by parents assumed that CBHWs possessed not only a good understanding of SRHR information (due to their professional role) but also a good understanding of ‘acceptable’ intergenerational and/or cultural norms around sexuality – just like traditional grandmothers.

*In the youth clubs, they’re also talked to by the female teachers, but we also attend, that’s when some of them [referring to adolescents] really pay attention when they realise you’re there…. the traditional advisor is there. – KII 14*.

Hence, in their work with adolescents, CBHWs often adopted the dual role of positioning themselves as both grandmothers and simultaneously as professional health workers. However, sometimes these two roles conflicted: for example, in relation to providing information about contraceptives to unmarried adolescents. The CBHWs navigated this conflict by providing ‘tailor-made’ information, depending on the audience and perceived audience needs. For example, they provided full SRHR information (including information related to access and use of contraceptives) but would emphasise abstinence. CBHWs only recommended condom and contraceptive use for sexually active adolescents. The quote below illustrates how one CBHW employed ‘tailor-made’ SRHR information when educating adolescents:

*We also teach children, the young people, we put them together and teach them the way they’re supposed to be living on sexual health issues…for girls who aren’t pregnant, we teach them “abstinence ili che” [abstinence is great], for those who are already having sex, we teach them not to have unprotected sex but to use condoms: (1) you won’t fall pregnant; and (2) you won’t contract diseases. Even me, I give them condoms; we get them from the clinic and give them out. But we don’t just give them, first we tell them. – KII 8*.

As can be seen in the above quote, the work of the CBHWs extended beyond being educators to encompass being service providers and a link to health services, as we describe in the next category.

#### Being service providers and a link to SRHR services

CBHWs also shared their experiences and perceptions of being service providers and at the same time a link to SRHR services, functions that are closely linked with providing education on SRHR. By providing SRHR services at the community level, CBHWs made these services easily available. Yet, due to their lack of medical training, the SRHR services they provided were primarily limited to condom distribution and counselling. One CBHW explained how she navigated her limited training by providing information to adolescents, encouraging abstinence, and only giving condoms when she considered that they ‘can’t abstain’:

*To the younger ones, I used to distribute information, telling them “you can protect yourself by using a condom if you find that you can’t abstain”… and I give them condoms when they ask for them because they’re free with me, they come and say “we need this and that”…they fear going to the clinic because some nurses shout at them: “you’re supposed to be at school” – KII 11*.

As the previous quote conveys, CBHWs considered themselves friendly and approachable (in contrast to the nurses at the clinic), which could be related to their role as grandmothers. Although the provision of condoms to unmarried adolescents could be understood as conflicting with the traditional grandmother’s role, most CBHWs willingly distributed condoms to adolescents whom they perceived to be in need of contraception and/or sexually active. This was considered a more favourable choice, with fewer negative outcomes than unprotected sex:

*…. You’ll find that they contracted these diseases, HIV, he/she is suffering, doesn’t look good but doesn’t go to the clinic. So that one will need to be helped [meaning escorted] so that he/she does go to the clinic, and we do help them. – KII 8b*.

Besides condom provision, CBHWs were viewed as key in providing other services, such as following up pregnant adolescents and linking them to antenatal care, as well as encouraging pregnant girls to continue their schooling. In performing this role, CBHWs were both caring and controlling (perhaps as grandmothers would be expected to be), as depicted in the following quote:

…*When the girl is pregnant, I won’t stop, that person is mine, I will even put them in my register and take them to the clinic. I will be following up and encouraging her that “once you deliver you have to go back to school”. – KII 3*.

Finally, and as illustrated in the previous quotes, to ensure that adolescents accessed other SRHR services and commodities that they could not provide, CBHWs also relied upon conducting escorted referrals to the health facilities. In all three quotes in this section, the participants described the need to escort adolescents to clinics, to ensure that they received the services they needed. In the quote from KII 8b, the role of escort is described as a crucial buffer against adolescents needing health services but not going to the clinic. This is related to the CBHW’s role of advocate, which we further describe in the next category.

#### Being advocates for adolescents and their SRHR

At the health facility level, escorted referrals for adolescents were not only undertaken to encourage adolescents to seek SRHR services, but also because CBHWs feared that some of the adolescents would fail to speak out if they were left to go alone and/or may not be treated well by the health workers. Hence, CBHWs took on the role of advocating on behalf of adolescents seeking contraceptives, as KII 13 describes:

*As a community health worker, I will go with the child that side [at the health facility] and as a community health worker I will be the one who talks with the nurses. Then I speak on behalf of the child, maybe she wants an injection… We [CBHWs] can stand on their behalf, and we’ve done it before. – KII 13*.

At the school level, CBHWs spoke on behalf of pregnant girls to teachers, the school authorities, and their fellow pupils, especially in situations where girls experienced stigma for attending school while pregnant. One CBHW described how she had helped a girl who was stigmatised for going to school while pregnant as follows:

*One child stopped going to school and we followed up and asked, “Why aren’t you going to class?” [She said:] “They laugh at me in class when they see me”, so we tried to talk to them…the madam [meaning the teacher and the pupils]. When they see her now, her friends are happy and laugh with her, she’s become free and plays with her friends. There’s nothing like her sitting gloomy, the children are free. – KII 14*.

In addition to advocating for adolescents at the health facility and school, CBHWs considered it their role to advocate on behalf of the adolescents with their parents/guardians, since they considered adolescents too young to discuss SRHR issues with their parents. Such an advocacy role was considered crucial for the prevention of early marriages. One CBHW explained how she talked parents out of marrying their children early.

*There are some parents who love money, so immediately a girl develops breasts they marry her off and get* lobola *[bridal money], so I go, sometimes with the village headman… and educate them on the benefits of education and not marrying their children early. I talk to the parents and tell them not to marry off the girls. – KII 5*.

And, to promote the return of girls to school after childbirth:

*Yes, I’ll go to her parents, the mother of the child, I’ll go and talk to her and tell her how it is and what the law says, that these days there is no child who is being forced to get married, we talk to parents, so they take care of the baby, then she [referring to the adolescent girl] can go back, here at school. – KII 4*.

Positioning themselves as grandmothers, and at the same time as CBHWs, made it possible for CBHWs to carry out this advocacy role. Playing this dual role equipped them with sufficient authority to approach everyone within the settings of the CBHS. As one participant explained:

*If we hear that someone wants to marry off their child, we go to visit them and ask if the child goes to school. [If] they reveal that it’s because of a lack of money, then we tell them they will be reported to the* induna *[headman]…so now we’ve been having meetings with the* induna *in the community…we tell them: “If we hear that you want to marry off your child, you’ll be fined a goat and you’ll look for money,” so that the case is taken to the chief. – KII 6*.

In playing their advocacy role, the CBHWs sometimes went beyond talking to parents to threatening them with reports and fines, including actually reporting to the headman those who refused to listen to the advice of not marrying off adolescents. In some cases of sexual violence, the CBHWs escalated the report to law enforcers, as outlined in the subsequent category.

#### Reporting sexual violence

In their role of reporting issues related to sexual violence, the CBHWs worked together with the police and other community-based law enforcers (such as headmen). Cases identified and reported by CBHWs included any form of sexual violence against adolescents, such as sexual harassment and other forms of sexual violence, including (but not limited to) rape.

When reporting sexual violence, CBHWs used their experience and knowledge coupled with expectations of both their grandmother and CBHW roles to interpret available by-laws and policies. In their traditional role of passing on intergenerational knowledge about sexuality, grandmothers were expected to include information on sexual violence against girls and women. However, the sexual violence information that grandmothers conveyed to adolescents and whether (or not) it conformed with expectations that CBHWs will report any form of sexual violence was not clearly defined by the participants. Furthermore, no standardized expectations or guidance were mentioned by the participants in relation to how they were supposed to handle and report sexual violence cases. For instance, they considered some cases of sexual violence to be less severe, while other cases were defined as severe. Sexual violence cases considered as less severe by CBHWs were not often reported to the police; rather, the CBHWs, together with community gatekeepers, negotiated with the perpetrator and only reported to the police if the perpetrator reoffended. In contrast, cases that were considered severe were reported immediately. The quotes below illustrate the reasoning of some CBHWs around reporting sexual violence:

*So, the child will just come straight… [to me] grandmother, “grandmother, this person has said this and this to me, has said bad things to me [referring to sexual harassment comments], they want to touch my breasts”. So, as a parent, you will go and talk to that person, if they continue or are stubborn, [you take them] straight to the police. – KII 1*.

I: The issue of rape?

*R: Yes, there’s a committee, I’m one of them, then there are others also, they’re also some from this school. There are about 10 of us. So, when there is that issue [referring to sexual violence], they report it to us…when we find that it’s serious, and it is true that things [sexual violence] happened, we report it to the police, that’s what we do. – KII Z*.

This last quote also describes how the CBHWs not only assessed the severity, but also judged whether ‘it is true or not’. However, the criteria employed for assessing whether sexual violence had occurred, and its severity, were not clearly defined. Some participants linked contraceptive and condom use with sexual violence, depicting victims of sexual violence as having the opportunity, choice, and responsibility to use condoms and contraceptives when subjected to sexual violence. For example:

…*we tell them: “these [condoms], it’s not that we’re giving them to you to promote sex, but if you are caught and harassed into having sex, you can use them”. We’re not giving you a leeway to use these condoms just anyhow. – KII 6*.

Despite these examples, it is important to highlight that most CBHWs considered sexual violence to be harmful and strived to ensure that perpetrators were reported, even if that meant going against the parents’ wishes. For example:

*Yes. If we find that it’s true, we report them [referring to the perpetrators of sexual violence] to the police, and they’re punished. We talk to the girls, and we talk to the parents, we tell the parents: “You’re not going to get…get the money, your child was raped, and we will report it to the police”. – KII 12*.

As shown in the quote above, CBHWs seemed to have substantial power in deciding what counts as ‘true’ sexual violence and how to proceed in each case.

## Discussion

This study has analysed how 14 CBHWs experienced their roles in addressing adolescents’ SRHR needs in rural Zambia. Our findings align with other studies in confirming that sociocultural norms pose challenges to CBHWs in the fulfilment of their different responsibilities [11, 22]. Furthermore, we have shown that CBHWs adopt a dual role of being part of the community (as a grandmother) and part of the health system (as a professional CBHW), to create/maximise opportunities and navigate challenges. This dual role was sometimes perceived as complementary, helping to promote openness, support, and care, while at other times the roles were perceived as conflicting with each other, especially in relation to navigating sociocultural expectations. These complementary and conflicting roles resulted in CBHWs employing discretion in how they provided SRHR information and services and how they advocated on behalf of adolescents.

### CBHWs as boundary spanners

Our findings portray CBHWs as individuals whose role requires them to operate across different arenas, bending borders and triggering collaboration between schools, the local healthcare facility, community leaders, and at times, the police. Boundary spanners are relevant and beneficial for delivering primary healthcare and reaching marginalised and hard-to-reach individuals [33, 35]. Our study highlights that adolescents’ SRHR programmes can benefit from CBHWs as boundary spanners on two levels: the community and the organisational level. At community level, CBHWs linked the adolescents to parents, while encompassing the sociocultural norms – and at times social expectations – using discretion, as we will also discuss in the next section.

At the organisational level, CBHWs linked adolescents in need of SRHR services with healthcare facilities, while those who dropped out of school were linked back to the school, and efforts were made to link adolescents to law enforcement in cases of sexual violence. Similarly to previous research, our study highlights that trust and respect are key to boundary spanning [33, 35]. In our case, CBHWs earned trust and respect from combining their grandmother and professional roles.

In the recent past, there has been a shift towards the use of peers to provide SRHR, with some authors reporting peer-led interventions as an effective health-promotion strategy for reaching adolescents with SRHR services and information [42–44]. However, there are also studies reporting that information from peers is less likely to be trusted by adolescents, in comparison to SRHR information obtained from grandparents and health workers [27]. 

In addition, hegemonic discourses that portray adolescents as immature, vulnerable, and in need of adult support could potentially disadvantage or limit adolescents’ ability to span organisational, symbolic, and social boundaries [33], while it may make this easier for adult actors, such as CBHWs (also considered as grandmothers in our study). A study by Menon showed that many young people reported being most comfortable when approaching their grandparents for credible SRHR information and advice [45], [46, 47]. 

CBHWs form a cadre of public workers who can play a key role in spanning boundaries related to adolescents’ SRHR services. In this study, the CBHWs who participated were not formally employed by the state but were linked to the local health facility and officially recognised as part of the CBHS – located at the intersection of various structures of the Zambian CBHS. In the context of this study, these structures include the lowest level of primary healthcare within the health system (the local health facility), but also the school, the police, and the community.

Despite being among the ultimate implementers of policy [2, 11, 13, 33, 48], CBHWs are largely invisible in policy discussions and implementation and are not considered to be at the same level as other public-sector workers, such as teachers or nurses [37]. Although CBHWs seem to have little power within the hierarchy of the CBHS’s structures (school, health facility, community, and police), their unique position at the intersections of these structures made them effective boundary spanners for supporting adolescents’ SRHR. In this intersectional but privileged position, CBHWs have access to expert knowledge and resources from the health facility and possess valuable knowledge about the micro-level dynamics of their community [37]. 

### CBHWs: navigating a dual role by exercising discretion

Our next point of departure is street-level bureaucracy, a theory that examines the day-to-day practices and behaviour of public workers such as civil servants, teachers, and nurses (referred to as street-level bureaucrats), who interact directly with citizens as they dispense or allocate social services. Street-level bureaucrats not only play a concrete role in policy implementation but also possess substantial discretion in the treatment of clients/users [29, 36, 38], and have considerable autonomy and decision-making power [37]. 

Similar to a study on the power of community health workers in health policy implementation in Brazil [37], our findings illustrate how, when providing SRHR education and services, CBHWs contribute to alleviating, reproducing, and/or exacerbating inequalities [49, 50]. For example, we found that exercising their grandmother role conferred upon CBHWs the legitimacy to provide SRHR education to both parents and adolescents. In this task, they both received support and were trusted to pass on intergenerational SRHR knowledge but were required to refrain from giving information about contraception use to unmarried adolescents. While not providing such information aligned with the grandmother’s role, it conflicted with expectations about what information the ‘professional CBHW’ should provide – which should include information about contraceptives. Trying to work around this conflict to ensure that both professional and community/social expectations were met, CBHWs exercised discretion by tailoring the information to each adolescent, depending upon what they thought each adolescent would need.

Training, resources, and the social, cultural, and political context can have serious implication for street-level bureaucrats and how they accomplish their roles [51, 52]. In this study, most of the CBHWs had no formal education to provide certain types of contraceptives and this limited the SRHR services that they could provide to adolescents. We assume that lack of formal training, coupled with socio-cultural norms and inadequate tools and SRHR commodities could leave CBHWs poorly prepared boundary spanners.

Zulu et al. summarise the three ways in which bureaucrats cope with challenges and dilemmas, by “adjusting or moving towards clients through bending policy options to meet the needs of clients; moving away from clients or rationing services; and moving against clients through rigid application of rules” [29, 37]. In our study, CBHWs moved towards adolescents and parents by ‘tailoring’ information, provision, and linkage to SRHR services. At the same time, we interpret their actions of restricting access to condoms and contraceptive for a certain group of adolescents as a way for CBHWs to move away from adolescents (clients) or rationalising SRHR services. Interestingly, this seemed to be a way for them to coping and adhering to social norms associated with the grandmother role rather than enforcing strict regulation, as Lipsky noted [36, 53] In our data, we did not find any evident pointing to CBHWs moving against adolescents (Lipsky’s third strategy) through the rigid application of rules.

This situation highlights the complexities the of CBHWs’ experiences in their dual role. Enhancing training for CBHWs to increase their knowledge and value clarification, coupled with the provision of adequate resources, could help improve their competency, service delivery and performance [26, 51]. 

Previous studies that have applied the theory of ‘street-level bureaucracy’ have recognised the autonomy and decision-making power that bureaucrats possess [22, 37, 38]. Such power often manifests in the form of domination. Power and domination may occur in three forms: domination that aims to shape behaviour through coercion/threats and defining what is correct and appropriate; domination as a productive force that aims to shape and govern capacities, competencies, and the will of the subject; and a hierarchical power that rewards and promotes certain behaviours and individuals while constraining others [37, 54]. 

Our findings highlight the ways in which CBHWs exercised domination in these three forms. Examples of domination exerted by our participants came in the forms of promoting the SRHR information and services which they defined as ‘correct and appropriate’ for adolescents and parents. In some instances, especially when parents were willing to push their children into early marriages, CBHWs resorted to using the threat of reporting such parents/guardians to the headman. Domination was also explicit in how CBHWs defined and judged sexual violence. We found it problematic that CBHWs used their own discretion in defining what was considered to be ‘severe or non-severe’ sexual violence because this could potentially mean that some cases of sexual violence were classified as non-severe and ignored. Equally, problematic is the potential for CBHWs classifying sexual violence reported by adolescents as ‘severe’ or ‘non-severe’ based on perceived truth, especially given that their relationship with adolescents relied on trust. It is possible that resolving what CBHWs perceived as less severe cases of sexual violence within the community instead of through the legal system could be considered a more effective approach to addressing sexual violence. This may be particularly true in situations where the legal system is mistrusted for their potential contribution to revictimization. As previous studies have shown, some of the barriers to reporting sexual violence or seeking care include poor responsiveness from law enforcement agencies, inability of the criminal justice to apprehend and prosecute the perpetrators, and discriminatory attitudes towards victims in courts and law enforcement settings [55, 56]. We argue that, considering the negative effects that sexual violence has on mental, physical, emotional, and sexual health [57], classifying any form of sexual violence as ‘non-severe’ could potentially lead to re-victimisation and the continuation of such violence. For adolescents, this may also mean trust and accessibility to supportive sexual violence services could be lost. More research is required to understand the effects of how sexual violence cases among adolescents are classified and reported in Zambia.

There is scarcity of studies on the role on CBHWS in reporting sexual violence. However, we agree with Gatugula et al., who reported that CBHWs frequently interact with sexually abused children, adolescents, and their caregivers. However, the lack of guidelines and protocols on how to report GBV coupled with lack of support, limited resources, security risk and social norms may hinder reporting of GBV by CBHWs [58]. Two strategies could be used to encourage the reporting of sexual violence by CBHWs. Firstly, deliberate efforts to ensure that CBHWs are equipped with clear information and adequate discussions about sexual violence among children and adolescents. Although not directly related to GBV, training has shown to improve the effectiveness of CBHW in delivering services [59]. Secondly, the presence of protocols and guidelines documenting how sexual violence should addressed could be another of the strategies to encourage the reporting of sexual violence [60]. Finally, ensuring efficient functioning of systems, treating adolescents reporting sexual violence respectfully, and offering non-judgmental support are vital for effective reporting [60]. This approach can contribute to empowering rather than re-victimizing individuals reporting incidents of sexual violence.

It is equally important to contextualise the power of CBHWs (whether they are powerful or powerless), in relation not only to their community but also to other street-level bureaucrats such as teachers, nurses, or doctors, who may be in more powerful positions. In fact, it is possible that CBHWs’ positioning at the lowest level of the pyramid of state bureaucrats may also mean that their power remains questionable, while nevertheless significant enough to influence how sexual violence is screened and reported.

### Limitations and strengths

To our knowledge, this is one of the first studies to analyse the role of CBHWs as boundary spanners and street-level bureaucrats within the context of the CBHS and in relation to adolescent SRHR. The literature provides evidence that many countries are currently aiming to promote adolescents’ SRHR through a variety of projects and using CBHWs to implement policy and deliver interventions [11, 30, 37]. Our study provides timely and relevant information to help guide the implementation of such projects.

Selecting participants from various communities allowed us to gain access to personal experiences and diverse perspectives. While recruiting only CBHWs who had previously participated in the SRHR project resulted in certain insights and experiences, we unfortunately lost the perspectives of CBHWs who had not participated in the SRHR project. It could be that those who had participated in the SRHR project have more positive experiences or where less critical towards SRHR interventions for young people.

To enhance the quality of qualitative data, it is imperative to establish rapport and foster mutual understanding between the researchers and the participants [61]. The interviews were conducted by the first author (CM), who although being an outsider from the study communities, had interacted with some CBHWs while collecting data for another study involving adolescents. This means that rapport was enhanced, but still the participants could perceive the interviewer as not directly involved with the intervention, allowing them more freedom to bring up criticism.

It was not originally intended to exclusively select female CBHWs, however, it was discovered that all the CBHWs who agreed to participate in this study were female. This outcome may be attributed to the initial goal of the SRHR project, which focused on empowering girls and tailored the interventions accordingly, and in accordance with socio cultural norms - conversations about sex tend to occur more frequently between women and girls, and between men and boys, with far less mixing between genders. Although boys in the schools were invited to attend the SRHR youth clubs and interacted with CBHWs, greater emphasis was placed on girls.

The perceptions obtained in our study was gendered with more emphasis on the girl’s sexuality. This could be because all the participants were female CBHWs. The fact that we got very little on the boys could mean that there is limited cross gender conversations on sexuality, or alternatively, it indicates that the burden of acquiring sexual education and information fall disproportionately on girls as noted in a separate study conducted by the authors [58]. By talking mainly about girls’ sexuality and directing their efforts towards girls CBHWs may be reinforcing that preventing negative sexual outcomes such adolescent pregnancy is a responsibility of girls.

It is also possible that some participants might have not been very critical in their responses hoping their positive assessment of the intervention could lead to more funding. To address this potentiality, a detailed explanation of study’s purpose was provided to all participants prior to their participation, explicitly stating that the study was not linked to future funding or employment. Meeting with CBHWs after the project had ended could have also allowed participant’s’ reflection without fear of losing project benefits. The nuanced nature of the responses in our study suggest CBHWs were willing to discuss their experiences, regardless of their prior involvement in the project.

Furthermore, the use of a grounded theory approach and the theoretical concepts of boundary spanning, and discretion enabled us to achieve a higher level of analysis and abstraction, which could enhance the transferability of our findings. However, our study was conducted within the context of a broader intervention designed to reduce teenage pregnancies and ensure that adolescents continue to attend school [39]. Hence, our findings may be of most relevance to settings with a similar context.

## Conclusion

Community-based health workers (CBHWs) play a crucial role in addressing adolescents’ SRHR needs. In undertaking their tasks, the dual roles of being both a grandmother and a professional CBHW were sometimes complimentary and at other times conflicting. CBHWs often resorted to discretion as a way of addressing the barriers, conflicts, and challenges they faced. More importantly, the findings from this study provide relevant evidence necessary for understanding how these key actors (CBHWs) in the Zambian CBHS can contribute to creating a system that is responsive to adolescents’ SRHR needs. Despite the challenges faced by CBHWs, they are the missing link to providing context specific and comprehensive SRHR information and services that could span all the boundaries in the CBHS. Thus, training should also equip them with the skills they need to navigate the increasingly complicated and complex contextual CBHS in which they operate.

### Electronic supplementary material

Below is the link to the electronic supplementary material.

Supplementary Material 1

## Data Availability

The data for this study can be accessed freely by sending a reasonable request to the corresponding author.

## Abbreviations

CBHS: Community-based health systems
CBHWs: Community-based health workers
ERES: Excellence in research ethics and science
RISE: Research initiative to support the empowerment of girls
SRH: Sexual and reproductive health
SRHR: Sexual reproductive health and rights
