# Supplementary material for: Being both a grandmother and a health worker: experiences of community-based health workers in addressing adolescents’ sexual and reproductive health needs in rural Zambia
Source: BMC Public Health. 2024 May 3;24:1228. doi: 10.1186/s12889-024-18685-6 (PMC11069181; doi:10.1186/s12889-024-18685-6)
Supplement: Supplementary file 1 — Supplementary Material 1 [file 12889_2024_18685_MOESM1_ESM.pdf]

Supplement 1: Interview Guide

Being both a grandmother and a health worker: Experiences of community-based health workers in addressing adolescents' sexual and reproductive health needs in rural Zambia

Interview Guide for sub-study 3

Aim of sub-study: To analyse the role of community-based health workers in developing a responsive community-based health system for adolescents in Zambia.

Participants: Community based health workers (CBHWs) and teachers who participated in the community component arm of the Research Initiative to Support the empowerment of girls (RISE)

Interview ID: ____

Name of community: ____

Age: ____

Sex: ____

Level of education: ____

Qualification (CBHW or Community health Assistant):______________________________

For how long have been working in this position: __________________________________

Background

1. Tell me bit about yourself. Who are you and what do you do? What qualifications do you have?
2. How long have you been working as a CBHW?

3. Why did you start working as a community health worker?
4. What type of work did you do with adolescents at community, facility, and school level before the RISE project?

Role in the community-based health system

Now I will ask you some questions about role developing a community-based health system that is responsive for adolescents. When I say community-based health system I'm referring to the community health-based workers or community health assistant working with the local health facility in addressing the sexual reproductive needs or issues that adolescents face.

1. What SHR issues do you think adolescents in your community face? Probe for school level, community level, facility level and family level.
2. How has your community-based health system been working to address these sexual reproduce problems that adolescents face? Probe: before RISE and After RISE
3. What role do you as you as community health workers or community health assistants been playing in making sexual reproductive health and rights form responsive or friendly in your community?

   Probe at the school; at the health facility and in the general community? Is your role the same before and after RISE?
4. To what extent do you think your community, the health facility and the schools are responsive or friendly to the sexual reproductive health of adolescents before the RISE project? If so, why? If not, why not?
5. What other things do you think can be done to ensure the community-based health system is friendly to adolescents?
6. What role do you think you or your fellow CBHW could play in making the SRHR services at your facility more responsive or friendly to adolescents?

a. How do you think community health workers can be a link between the adolescents and: 1) the health facility 2) school and 3) the community?

7. How did the RISE project influence your role as a CBHW? Did it help you to change the way you deal with adolescents in your community? If yes, how? If not, why not?
8. Whom do you work with to achieve your roles? Probe on how they work with the mentioned people.
9. Who else apart from the people you've mentioned do you think are key in supporting you perform your role? How are they key and why do you think they are key?
10. Do you think adolescents would be one of the key people in making the community-based health system more responsive or friendly to their sexual reproductive needs? If yes, why do you think so? If no, why?
11. Are there other community-based health workers in your community who work to improve the sexual reproductive health of adolescents in your community? What exactly do they do? And why do you think their work would make the community-based health system more friendly to the sexual reproductive health and rights of adolescents?
12. Apart from the roles that you have mentioned above, what do you think are some of the other things that CBHWs in your community can do to make the community-based health system more responsive to adolescents?
13. Informal chat about the programme theory (developed in sub-study 1) and the end of the short term SRHR intervention.

**Programme Theory 1**	**Programme Theory 2**

**IF** interventions that aim to address the SRHR issues of adolescents in the CBHS are supported by existing national policies, socio-cultural norms, existing CBHS structures and functioning local health facilities.<br>**THEN** the interventions are likely to make all key stakeholders in the CBHS develop a positive attitude towards adolescents' SRHR, which will encourage uptake of SRHR services and information.<br>**BECAUSE,** at individual level, the intervention will improve awareness and knowledge, clarify the values on SRHR among all the actors (adolescents, CBHWs, teachers and community members), which will at collective level lead to improved communication and cohesion, social connections and linkages among the actors**.** **AS A RESULT**, the 'ordinary' CBHS will be transformed into a CBHS that is responsive to the SRHR needs of adolescents	**IF** interventions that aim to address the SRHR issues of adolescents in the CBHS are NOT supported by the existing national policies, rejected due to socio-cultural norms and not integrated in the existing CBHS structures and functional local health facilities.<br>**THEN** the interventions are likely to make all the key stakeholders develop or maintain a negative attitude towards adolescents' SRHR, which will discourage uptake of SRHR services and information.<br>**BECAUSE** at individual-level the intervention will NOT improve awareness and knowledge, clarify the values on SRHR among all the actors (adolescents, CBHWs, teachers and community members) and at a collective level the intervention will not lead to improved communication and cohesion, social connections and linkages among the actors. **AS A RESULT,** the 'ordinary' CBHS will NOT be transformed into a CBHS that is responsive to the SRHR needs of adolescents

Thank you very much for spending this time discussing with me. The information you have given me is very valuable. Do you have anything else to say in addition to what you have already said before we close?

End
